# Insights from a Nine-Segment Biomechanical Model and Its Simulation for Anthropometrical Influence on Individualized Planche Learning and Training in Gymnastics

**DOI:** 10.3390/bioengineering10070761

**Published:** 2023-06-25

**Authors:** Xiuping Wang, Gongbing Shan

**Affiliations:** 1 Department of Physical Education, Xinzhou Teachers’ University, Xinzhou 034000, China; wangxp@xztu.edu.cn; 2 Biomechanics Lab, Faculty of Arts & Science, University of Lethbridge, Lethbridge, AB T1K 3M4, Canada

**Keywords:** variations of body type, personalized motor control, gender, race

## Abstract

The Planche is a challenging, the most required, and a highly valued gymnastic skill. Yet, it is understudied biomechanically. This article aims to explore the anthropometric variations that could affect the quality of balancing control in the Planche and to identify the body types that have an advantage in learning and training. To achieve this goal, a 9-segment rigid-body model is designed to simulate the skill performance by using 80 different body types. The results demonstrate that body type is a critical factor in determining an individual’s innate ability to perform the Planche. The innate ability is affected by body mass, height, gender, and race. The findings reveal that a personalized training plan based on an individual’s body type is necessary for optimal learning and training. A one-size-fits-all approach may not be effective since each individual’s body type varies. Additionally, this study emphasizes the importance of considering segmental and/or limb characteristics in designing effective training plans. This study concludes that, for a given height, individuals with relatively longer legs and a shorter trunk (the characteristics of Europeans in comparison to Asians) could be better suited to perform the Planche. This suggests that European body types are naturally more advanced than Asian body types when it comes to performing the Planche. The practical implications of the current study are that practitioners can use biomechanical modeling and simulation techniques to identify body types that are most suited for the Planche and design training programs that are tailored to individual body types for optimizing their learning and training.

## 1. Introduction

The Planche is one of the most iconic and challenging movements in gymnastics. It involves supporting the body in a horizontal position with the arms extended in front of the body and the feet off the ground. This requires power, stability, and control, making it a critical skill for gymnasts to master. According to the Fédération Internationale de Gymnastique (FIG), the Planche is included in the “Static Strength Elements” category and is often performed in men’s floor exercise, rings, parallel bars, and pommel horse competitions, as well as in women’s balance beam and floor exercise competitions [1]. Therefore, it is one of the most required skills in gymnastics.

From a motor control perspective, the Planche requires exceptional upper body strength, particularly in the shoulders, arms, and core muscles [2]. This strength is essential for other gymnastic skills, such as the handstand and iron cross on rings. Mastering the Planche also helps to develop the athlete’s overall body control and balance. Moreover, the Planche is a skill that is highly valued in gymnastic competitions [1]. It requires a high degree of technical proficiency and artistry, making it a skill that is both challenging and aesthetically impressive. Hence, the Planche is an essential skill for gymnasts to master.

Practically, it is known that the Planche is a high-level skill that requires years of dedicated training to master [2]. From the principles of biomechanics [3], the Planche requires static strength and accurate control in the shoulders and wrists. Proper technique, body alignment, and balance are essential to performing the Planche safely and effectively. As such, the knowledge obtained from biomechanics studies should be incorporated into Planche learning and training to provide targeted exercises and practice to help athletes increase learning/training efficiency in order to achieve mastery of this skill effectively [4,5]. Yet, a search using the keywords “Planche + biomechanics” in Web of Science in March 2023 returned zero records. Obviously, the Planche is understudied biomechanically. As such, biomechanical fundamental studies are needed to identify the key parameters that contribute to a successful performance. Understanding these factors could improve the efficiency and effectiveness of skill learning and training. Therefore, the relevance of studying Planche biomechanics lies in the potential to improve training methods in gymnasts [4,5].

One fundamental type of study in biomechanics is modeling and simulation, which enables researchers to investigate aspects that are typically difficult to measure or invisible to the naked eye. In the past few decades, Yeadon and his team have conducted numerous studies that have demonstrated the value of biomechanical modeling and simulation in enhancing gymnastic performance [6,7,8,9,10]. Yeadon’s investigations have yielded novel insights into gymnastic motor skills, advanced training methodologies, performance optimization, and the discernment of the degree of control involved in complex gymnastic movements. His research efforts have substantially aided researchers and coaches in expanding their understanding of this acrobatic discipline. These insights have not only facilitated improved training techniques for gymnasts but have also provided essential knowledge on how to acquire some complex gymnastic skills efficiently. In short, by comprehending the underlying mechanisms of motor skills through biomechanical modeling and simulation, researchers and coaches can design more effective training programs tailored to individual requirements while optimizing performance outcomes. Therefore, the biomechanical modeling and simulation technology was applied in the current study.

As stated above, the Planche is dominated by static balancing [1,2]. The kinetic mechanism of the static balancing has been extensively elaborated in biomechanical textbooks [3,4]. It can be quantitatively determined through the application of a 2D biomechanical model with rigid segments. Nevertheless, an essential aspect that remains overlooked is the anthropometrical influence on the learning and training of the static balancing. The relationship between gymnastic performance and anthropometry (i.e., the body type that is often characterized by using body mass and body height, such as heavy/tall or light/short) has been studied extensively. Previous research consistently indicates that body mass and height have significant implications for biomechanical optimization in gymnastic performance [11,12,13]. Further, other anthropometrical characteristics of an athlete’s body, such as the segmental and/or limb lengths, can also significantly affect their motor skills [14,15,16]. Consequently, body types and segmental characteristics will affect the execution of advanced gymnastic skills, such as the Planche. Without fundamental knowledge, the efficiency and effectiveness of training the Planche will be negatively influenced. Clearly, there is a gap in the current knowledge for learning the Planche in coaching practices.

This article aims to explore the anthropometrical variations that could affect the quality of control and balancing in the Planche, with a specific focus on identifying the body types that have an advantage in the learning/training by using a specially designed nine-segment rigid-body model and its simulations. The goals are to provide a comprehensive overview of the anthropometrical factors underlying the successful performance of the Planche and to highlight areas for future research. Ultimately, this study aims to provide practical knowledge for practitioners to develop more effective training methods for learners performing this demanding skill.

## 2. Materials and Methods

**Figure 1:**
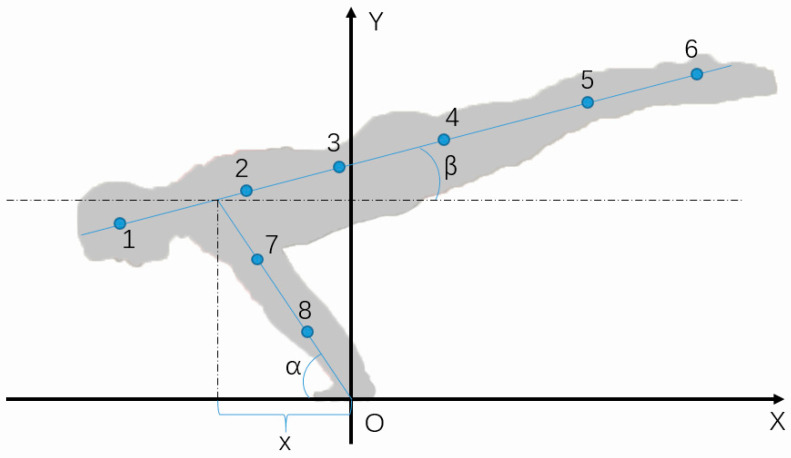
The 2D 9-segment biomechanical body model of the Planche. The blue dot: segmental center of gravity; *x*: the horizontal coordinate of shoulders.

The present study investigates the anthropometric influences on learning the Planche through biomechanical modeling and simulations. Previous research has established the utility of a 15-segment full-body biomechanical model for analyzing athletes’ movements to determine optimal techniques for various sports skills [17,18,19,20,21]. However, due to the Planche’s unique symmetrical characteristic in the medial–lateral direction, a 2D 9-segment model is developed (Figure 1). By merging the limb segments, the simplified 9-segment model includes the head, upper trunk, lower trunk, thighs, shanks, feet, upper arms, lower arms, and hands. The accurate control of the shoulders and wrists has been identified as the key control parameter influencing the skill quality [2], which can be quantified by the segment angles *β* and *α* in the 9-segment model, respectively. The validity of the static model used in this study is mechanically well proven [3,4].

Anthropometric factors, including body mass (BM), body height (BH), gender, and race, are important in determining human body size and shape. BM and BH have been proven to be the determinants of the absolute segmental masses and lengths [15,22,23], while gender and race influence the relative segmental masses and lengths [15,23]. In other words, for the same body mass and height, different genders and/or races possess different segmental masses and lengths. Regarding Planche performance, Figure 1 suggests that, mechanically, the lengths of the trunk, legs, and arms should play important roles. A previous study has clearly indicated the differences in these lengths due to gender and race (Table 1). Hence, 4 race–gender groups, namely, Asian–male, Asian–female, European–male, and European–female, are selected for the current study.

Combinatorially, the four anthropometric factors (BM, BH, gender, and race) fundamentally affect human motor learning and are critical in developing personalized training programs [11,12,13]. Numerous studies have established regression equations to quantify the influence of these factors on segmental characteristics, such as the Hanavan model [22], which quantifies the influence of BM and BH, and the Zatsiorsky model [23], which considers BM, BH, and gender. However, only one study [15], based on Web of Science, has quantified the influence of BM, BH, gender, and race on segmental characteristics. Therefore, the regression equations from this study are utilized to build the 9-segment model and perform the simulations to identify appropriate control characteristics for various body types.

In athletic training, body type classification is relevant [11,14,23]. The selection of body types should be based on BM, BH, gender, and race. An accurate classification is essential, as BM and BH are key factors in determining the absolute segmental masses and lengths, while gender and race result in differences in the relative segmental masses and lengths. To determine the influence of body-type variations on learning/training the Planche, simulations are performed using the Simulink software supplied in MATLAB [24]. The segmental masses, segmental lengths, and segmental center of gravity (COG) are determined using regression equations established in the previous study [15]. The torques of each segment are determined by using the following equations:

(1) $$T_{1}=\left[x-\left(L_{1}-D_{1}\right)\cos\beta \right]m_{1}g$$

(2) $$T_{2}=\left(x+D_{2}\cos\beta \right)m_{2}g$$

(3) $$T_{3}=\left[x+\left(L_{2}+D_{3}\right)\cos\beta \right]m_{3}g$$

(4) $$T_{4}=\left\{x+\left[L_{2}+L_{3}+\left(L_{4}-D_{4}\right)\right]\cos\beta \right\}m_{4}g$$

(5) $$T_{5}=\left\{x+\left[L_{2}+L_{3}+L_{4}+\left(L_{5}-D_{5}\right)\right]\cos\beta \right\}m_{5}g$$

(6) $$T_{6}=\left\{x+\left[L_{2}+L_{3}+L_{4}+L_{5}+\left(L_{6}-D_{6}\right)\right]\cos\beta \right\}m_{6}g$$

(7) $$T_{7}=\frac{xm_{7}g\left(D_{7}+L_{8}\right)}{L_{7}+L_{8}}$$

(8) $$T_{8}=\frac{xm_{8}gD_{8}}{L_{7}+L_{8}}$$

where *T_i_*: segmental torque, *x*: the horizontal coordinate of shoulders, *m_i_*: segmental mass, *L_i_*: segmental length, *D_i_*: the location of segmental COG, and *g*: gravity.

Mechanically, the equation below

(9) $$\sum_{i=1}^{8}T_{i}=0$$

governs the Planche equilibrium and is applied in the simulations of this study. Anatomically, ∣*x*∣ should be smaller than *L*_7_ + *L*_8_. Therefore, if a simulation provides a solution where ∣*x*∣ is larger than *L*_7_ + *L*_8_, it is considered as having no solution; i.e., the Planche is impossible for the selected body type. For all possible Planches, *α* is calculated by using the formula below:

(10) $$\alpha =180-\cos^{-1}\frac{x}{L_{7}+L_{8}}$$

Simulations are conducted on the age group of 12–20 years old. The preliminary selection ranges for BM and BH are selected based on the 95th percentile established in Chinese and German norms [25,26] for both race groups; then, the common range of both racial groups is finally used for the classification of body types. The BM classification is determined by equally dividing the finally selected range (i.e., the common range of both racial groups) into five weight classes (rounded up to the nearest integer), while the finally selected range of BH is equally divided into four height groups (Table 2). Consequently, there are 20 variations of body type for each race–gender group, i.e., Asian–male, Asian–female, European–male, and European–female. In total, the simulation study includes 80 variations to quantify the influence of BM, BH, gender, and race on Planche performance.

The model simulations in Simulink are organized with automatic inputs for all anthropometrical and kinetic calculations. Specifically, the inputs of Equations (1)–(8) are the anthropometrical data obtained via the regression equations using BM, BH, race, and gender [15]. The body horizontal angle *β* is the input of Equation (9) for calculating the shoulder horizontal position *x*, whereas *x* is the input of Equation (10) for obtaining the arms’ angle *α*. In short, the simulations provide the control characteristics of the body horizontal angle *β* and the arms’ angle *α*, as shown in Figure 1, for a quantitative analysis based on the selected body types.

The Fédération Internationale de Gymnastique (FIG) has set strict rules on angle *β*; for example, “the body line must not exceed 20° above parallel” [27]. As a result, the biomechanical simulations in the current study set the range for *β* to 0–20°. Under this condition, the wrist angle *α* can be used to analyze the advantage/disadvantage of different body types for performing the Planche; i.e., as *α* decreases, the difficulty rate for maintaining proper balance will increase.

## 3. Results

**Figure 2:**
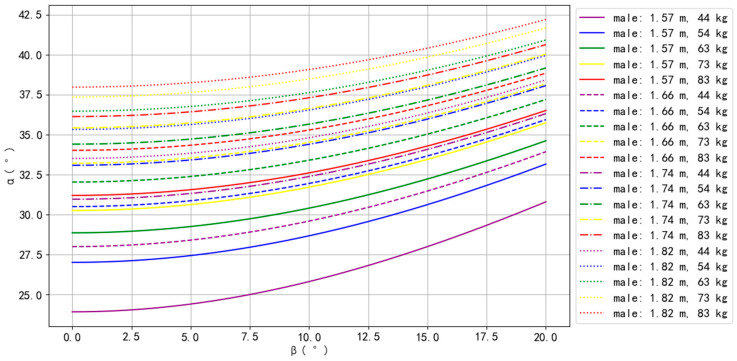
The simulation results of Asian males.

**Figure 3:**
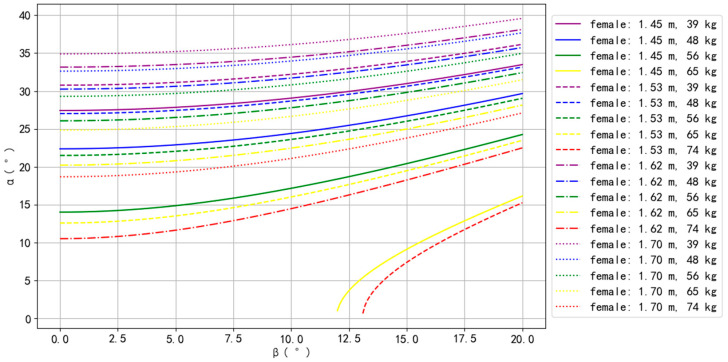
The simulation results of Asian females.

**Figure 4:**
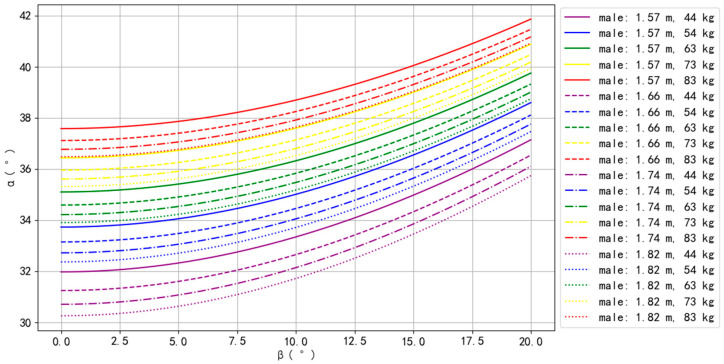
The simulation results of European males.

**Figure 5:**
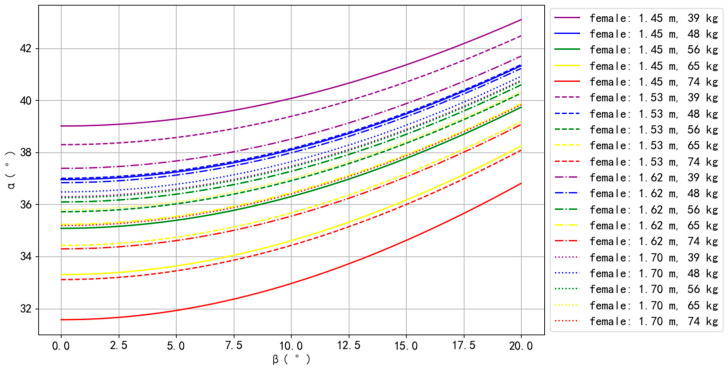
The simulation results of European females.

The simulation results for Asian–male, Asian–female, European–male, and European–female are presented in Figure 2, Figure 3, Figure 4 and Figure 5.

For Asian–male, the body types that could perform the Planche more easily were 1.82 m with 83 kg, 73 kg, and 63 kg, while the body types that could perform the Planche more difficultly were 1.57 m with 44 kg and 54 kg, as well as 1.66 m with 44 kg. For all body types, *α* increased as *β* increased. Furthermore, for the same BH, *α* increased as BM increased; equivalently for the same BM, *α* increased as BH increased (Figure 2).

For Asian–female, only one body type could not perform the Planche, with a BH of 1.45 m and BW of 74 kg. For the remaining body types, the top three that could perform the Planche more easily were 1.70 m with 39 kg, 1.62 m with 39 kg, and 1.70 m with 48 kg, while the bottom three that could perform the Planche more difficultly were 1.53 m with 74 kg, 1.45 m with 65 kg, and 1.62 m with 74 kg. Similar to Asian males, *α* increased as *β* increased, and, for the same BM, *α* increased as BH increased. Contrary to Asian males, for the same BH, *α* decreased as BM increased (Figure 3), indicating that increasing weight may make balancing more difficult.

For European–male, the body types that could perform the Planche more easily were 1.57 m with 83 kg, 1.66 m with 83 kg, and 1.74 m with 83 kg, while the body types that could perform the Planche more difficultly were 1.82 m with 44 kg, 1.74 m with 44 kg, and 1.66 m with 44 kg (Figure 4). For all body types, *α* increased as *β* increased. Furthermore, for the same BH, *α* increased as BM increased, but for the same BM, *α* decreased as BH increased, indicating that increasing body height may make balancing more difficult.

For European–female, the top three body types that could perform the Planche more easily were 1.45 m with 39 kg, 1.53 m with 39 kg, and 1.62 m with 39 kg, while the bottom three that could perform the Planche more difficultly were 1.45 m with 74 kg, 1.53 m with 74 kg, and 1.45 m with 65 kg (Figure 5). *α* increased as *β* increased, and, for the same BM, *α* increased as BH increased, but for the same BH, *α* decreased as BM increased.

**Figure 6:**
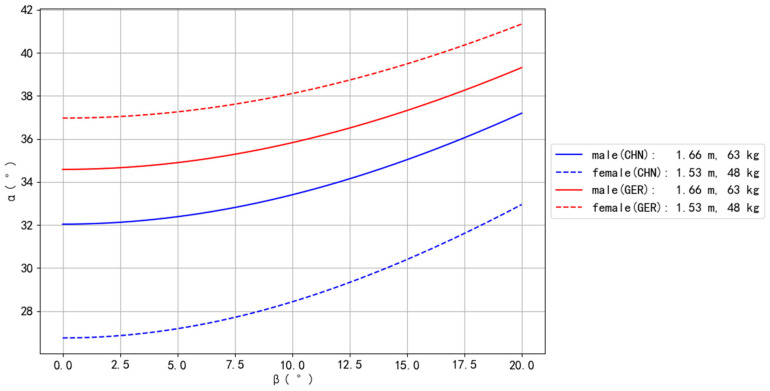
The influences of gender and race on Planche performance.

Comparing the average BM and BH of Olympic male (1.66 m, 63 kg) and female (1.53 m, 48 kg) gymnasts [28], this study found that European gymnasts can perform the Planche more easily than Asian gymnasts. Moreover, European female gymnasts can perform the Planche more easily than European male gymnasts, while Asian male gymnasts can perform the Planche more easily than Asian female gymnasts. The body type of European female gymnasts was found to be the most suitable for performing the Planche, followed by that of European male, Asian male, and Asian female gymnasts. These results are presented in Figure 6.

## 4. Discussion

The present study used biomechanical modeling and simulations to investigate the effects of body type on the performance of the Planche, one of the most required gymnastic skills that needs a high degree of upper body strength, balance, and coordination. The simulation results demonstrated that body type is a critical factor in determining the natural competence of an individual to perform the Planche, and this innate characteristic is affected by body mass, body height, gender, and race. A general interpretation of the simulation findings can be found in Table 3. The findings have practical implications for gymnastic coaches and trainers, as detailed below.

Firstly, the inherently advantaged body types for Asian males were found to be tall and heavy/normal weight, while tall and light body types were advantageous for Asian females. For European males, short/normal height and heavy body types were considered naturally advantaged, whereas for European females, short and light body types were advantageous. These results clearly indicate that individual body type plays a crucial role in determining one’s innate ability to perform the Planche. The results reveal that a one-size-fits-all approach to Planche training may not be effective, and a personalized training plan based on an individual’s body type may be necessary for optimal learning and training. As revealed by a recent systematic review article on anthropometry and competitive performance in gymnastics, the appearance of body shape has a significant impact on the evaluations of the execution of gymnastic movements in rhythmic, acrobatic, and aesthetic gymnasts [29]. The present study further highlights that body type is an innate determinant for executing gymnastic skills. This innate factor plays an important role in coaching practice, because it is well known that body type is highly individualized. Overall, our study underscores the importance of personalized training plans that consider individual body types in optimizing gymnastic learning and training.

Secondly, the challenge of body type variations induced by weight, height, gender, and race can be easily addressed through the use of biomechanical modeling and simulation techniques. By utilizing these tools, trainers and coaches can identify the body types that are the most suitable for the Planche and design training programs that optimize performance based on each athlete’s unique body type. This study demonstrates that the method used in this investigation is able to analyze an individual’s innate factors relevant to the optimal training regimen for that individual. Such a training plan is tailored to an individual’s specific physical characteristics. By incorporating these factors into training, individuals can achieve their goals in a more efficient and effective manner.

Thirdly, for a given height, individuals with longer legs and shorter upper bodies could be more suited to perform the Planche; i.e., individuals with a higher center of gravity (COG) may have an advantage in learning the Planche than individuals with a lower COG. Previous studies have revealed that, under the same BM and BH conditions, European people have a shorter trunk and longer legs (i.e., a higher COG) than Asians [15]. Regarding gender, for the same BM and BH, European females have a higher COG than males [15,23], while the reverse result is found among Asians [15]. These results consistently support our findings (Figure 6). Specifically, European female gymnasts are the most suitable for performing the Planche, while Asian female gymnasts have the most difficulty in learning the Planche. These results highlight the impact of race on the innate ability to perform the Planche, with European body types being more naturally advantaged than Asian body types. These results have additionally led to an anthropometrical post-exploration aiming to determine whether the relative body structure (i.e., the length ratios of segments) could be used as easy and practical indicators for selecting advantageous body types in learning the Planche. The present findings imply that three segments, namely, the trunk, legs, and arms, would be suitable candidates for the post-explorations. This is because the length ratios of the trunk and legs are related to the relative COG height, while the relative length of the arms is associated with the wrist angle *α*, used to analyze the difficulty rate of maintaining proper balance in the Planche. Hence, the length ratios of the trunk/leg, trunk/arm, and/or arm/leg were post-investigated. The ratios were obtained by performing regression equations using data from young German and Chinese subjects [15]. The means and standard deviations were calculated based on 20 body types in each of the four race–gender groups. The results of the post-exploration are shown in Table 4.

The anthropometrical post-exploration revealed that Asian females have relatively longer trunks and shorter legs (91.9%), while European females have less extreme ratios (83.0%). In contrast, European males have the shortest trunks relative to their legs (77.0%). Moreover, the length ratios of the trunk/arm influence the easiness of the Planche position. Asian females have the longest trunks compared to their arms (151.4%), followed by Asian males (134.4%), European females (132.4%), and European males (127.8%). The length ratio of the arm/leg may not play a significant role in Planche performance, but the result shows that European females have relatively longer arms (62.6%) than other groups (Table 4).

However, none of the length ratios in the post exploration showed an identical result to the simulation related to race–gender in Figure 6, indicating that no simple ratio parameter exists for selecting the proper body type for learning the Planche. Nonetheless, the ratio difference between the trunk/arm and trunk/leg (the fifth column in Table 4) demonstrates the same ranking order (i.e., European female > European male > Asian male > Asian female) as shown in Figure 6. This analysis may suggest that the two length ratios could combinatorially determine the advantage of body type in learning the Planche. Consequently, it may indicate that a user-friendly method could be developed for coaching practice. Of course, more studies are needed to confirm the practicality of this approach for body type selection and/or evaluation.

This is the first biomechanical study that quantitatively explores the influence of body type on learning and training the Planche. It is understandable that there are limitations associated with this study. The obvious one is the lack of consideration of causal factors, such as the role of muscle strength in learning/training the Planche. As elaborated in the current paper, the Planche is an advanced gymnastic skill that requires a high degree of upper body strength. As such, the contribution of the current study is limited to anthropometry only. Mechanically, it is well known that tall and/or heavy individuals need more muscle strength to perform the Planche, and, empirically, this is proved by the average short body height of elite gymnasts [28,30]. Therefore, the purely anthropometric results obtained from the current study, such as the advantageous body type for Asian males being tall and heavy/normal weight, may not be realistic. A logical hypothesis is that the balancing ability of tall and heavy individuals is limited by their muscle strength. Biomechanical dynamic modeling can be used to verify this hypothesis [31,32]. The second limitation is related to the application range of the regression equations employed for the anthropometrical data simulation. The regressions [15] are established based on young German and Chinese subjects aging from 15 to 26 years old; therefore, the simulation results may have large deviations for learners under 15 years old or for Southern Europeans or Middle Eastern Asians. In order to obtain accurate and individualized feedback, a proper anthropometric databank or database is advised for an individual model simulation. The third limitation is that this study is restricted to a simulation-based approach and does not include real-world testing of the Planche. Realistically, some of the selected body types in the simulations may not be able to perform the skill. Hence, future studies can extend the current study to include motion capture technology to carry out real-world testing of the Planche in individuals with different anthropometric characteristics. Additionally, dynamic modeling can also provide valuable information on the prevention of muscle injuries during learning and training [31,32]. Therefore, the insights from both innate and causal factors will provide a holistic picture for practitioners to design their individualized training. Future studies may try this avenue (i.e., motion capture and biomechanical modeling) for revealing more insights related to individualized training. In short, as an initial biomechanical study on Planche learning, the current study provides only a fundamental view for practitioners. 

## 5. Conclusions

In conclusion, this study contributes to the understanding of the relationship between body type and Planche performance in gymnastics. An individualized approach to training should be the most effective for improving the performance of the Planche. By considering an athlete’s specific anthropometric characteristics and developing training programs that target their unique needs, coaches and trainers would be able to help gymnasts improve their Planche performance and reach their full potential. Further research is needed to investigate the effects of causal factors, as well as the joint effects of innate and causal factors, on Planche performance to establish a science-based effective learning/training program.

## Figures and Tables

**Table 1:** The influence of gender and race on the relative segmental lengths (% BH) of trunk, legs, and arms under the condition of the same BM and BH (The data are from Shan’s study published in *Applied Ergonomics* [15]).

Segment	Gender	Asian	European
Trunk	Male	39.53	37.92
	Female	40.19	38.54
Legs	Male	48.15	49.83
	Female	47.29	49.01
Arms	Male	40.46	41.43
	Female	39.21	41.51

**Table 2:** The selected body types for biomechanical model simulations.

Body Type	Male	Female
Body Weight (kg)	44	39
	54	48
	63	56
	73	65
	83	74
Body Height (m)	1.57	1.45
	1.66	1.53
	1.74	1.62
	1.82	1.70

**Table 3:** The inherently advantaged and disadvantaged body types in learning the Planche.

Race–Gender	Naturally Advantaged Body Type	Naturally Disadvantaged Body Type
Asian–male	Tall and heavy/normal weight	Short and light
Asian–female	Tall and light	Short and heavy
European–male	Short/normal height and heavy	Tall/normal height and light
European–female	Short and light	Short and heavy

**Table 4:** Post-explorations related to the possibilities for developing user-friendly parameters for selecting body types in learning the Planche.

Race–Gender	TL/AL (%)	TL/LL (%)	AL/LL (%)	TL/AL–TL/LL
Asian–male	134.4 ± 5.1	82.7 ± 2.4	61.5 ± 1.0	51.8 ± 3.0
Asian–female	151.4 ± 20.0	91.9 ± 12.5	60.7 ± 1.0	59.5 ± 7.7
European–male	127.8 ± 1.5	77.0 ± 0.9	60.2 ± 0.7	50.9 ± 1.3
European–female	132.4 ± 6.6	83.0 ± 7.5	62.6 ± 2.7	49.4 ± 1.7

TL: trunk length, AL: arm length, LL: leg length.

## Data Availability

Not applicable.

## References

[1] FIG (2022). The Official Rulebook of International Gymnastics Federation. https://www.gymnastics.sport/site/rules/.

[2] Katrichis N.E., Moca A. (1992). Sports performance series: The planche. Strength Cond. Assoc. J..

[3] Hay J. (1978). The Biomechanics of Sports Techniques.

[4] Ballreich R., Baumann W. (1996). Grundlagen der Biomechanik des Sports (The Basics of Biomechanics in Sports).

[5] Shan G., Sust M., Simard S., Bohn C., Nicol K. (2004). How can dynamic rigid-body modeling be helpful in motor learning? Learning performance through dynamic modeling. Kinesiology.

[6] Yeadon M. (1993). The biomechanics of twisting somersaults. Part I: Rigid body motions. J. Sport. Sci..

[7] Yeadon M., Hiley M. (2000). The mechanics of the backward giant circle on the high bar. Hum. Mov. Sci..

[8] Yeadon M., Knight J. (2012). A virtual environment for learning to view during aerial movements. Comput. Methods Biomech. Biomed. Eng..

[9] Yeadon M., Hiley M. (2014). The control of twisting somersaults. J. Biomech..

[10] Yeadon M., Hiley M. (2018). The limits of aerial techniques for producing twist in forward 1½ somersault dives. Hum. Mov. Sci..

[11] Zhang X., Shan G., Wang Y., Wan B., Li H. (2019). Wearables, biomechanical feedback, and human motor-skills’ learning & optimization. Appl. Sci..

[12] Altavilla G., Di Tore P.A., Lorenzo R., Tiziana D.I. (2017). Anthropometric, physiological and performance aspects that differentiate male athletes from females and practical consequences. J. Phys. Educ. Sport.

[13] Wąsik J., Ortenburger D., Góra T., Shan G., Mosler D., Wodarski P., A Michnik R. (2018). The influence of gender, dominant lower limb and type of target on the velocity of taekwon-do front kick. Acta Bioeng. Biomech..

[14] Winter D.A. (2009). Biomechanics and Motor Control of Human Movement.

[15] Shan G., Bohn C. (2003). Anthropometrical data and coefficients of regression related to gender and race. Appl. Ergon..

[16] Zatsiorsky V. (2008). Biomechanics in Sport: Performance Enhancement and Injury Prevention.

[17] Shan G., Westerhoff P. (2005). Full body kinematic characteristics of the maximal instep Soccer kick by male soccer players and parameters related to kick quality. Sport. Biomech..

[18] Yu D., Yu Y., Wilde B., Shan G. (2012). Biomechanical characteristics of the Axe Kick in Tae Kwon-Do. Arch. Budo.

[19] Zhang Z., Li S., Wan B., Visentin P., Jiang Q., Dyck M., Li H., Shan G. (2016). The Influence of X-Factor (Trunk Rotation) and Experience on the Quality of the Badminton Forehand Smash. J. Hum. Kinet..

[20] Shan G., Visentin P., Zhang X., Hao W., Yu D. (2015). Bicycle kick in soccer: Is the virtuosity systematically entrainable?. Sci. Bull..

[21] Liu Y., Kong J., Wang X., Shan G. (2020). Biomechanical analysis of Yang’s spear turning-stab technique in Chinese martial arts. Phys. Act. Rev..

[22] Hanavan E.P. (1964). A Mathematical Model of the Human Body.

[23] Zatsiorsky V. (1983). The mass and inertia characteristics of the main segments of the human body. Biomech. IX.

[24] (2020). MathWorks, Simulink V9.9.

[25] SAC (1989). Human Dimensions of Chinese Adults. https://std.samr.gov.cn/gb/search/gbDetailed?id=71F772D78375D3A7E05397BE0A0AB82A.

[26] Neuhauser H., Schienkiewitz A., Rosario S.A., Dortschy R., Kurth B.-M. (2016). Reference Percentiles for Anthropometric Measures and Blood Pressure Based on the German Health Interview and Examination Survey for Children and Adolescents 2003–2006 (KiGGS). https://edoc.rki.de/bitstream/handle/176904/3271/23s3ntHQtKbus.pdf?sequence=1.

[27] FIG (2019). 2021—2024 Code of Points. https://aerobicwiki.de/content/6-tk-aerobic/3-archiv/20210131-aerobic-tk-neuer-code-of-points/aer_2021-2024_cop-draft-september-2019-for-nf_s.pdf.

[28] Atikovic A. (2020). Anthropometric Characteristics of Olympic Female and Male Artistic Gymnasts from 1996 to 2016. Int. J. Morphol..

[29] Kaur K., Koley S. (2019). Anthropometric determinants of competitive performance in gymnastics: A systematic review. Int. J. Health Sci. Res..

[30] Sands W.A., Slater C., McNeal J.R., Murray S.R., Stone M.H. (2012). Historical Trends in the Size of US Olympic Female Artistic Gymnasts. Int. J. Sport. Physiol. Perform..

[31] Prassas S.G. (1988). Biomechanical Model of the Press Handstand in Gymnastics. Int. J. Sport Biomech..

[32] Wan B., Shan G. (2016). Biomechanical modeling as a practical tool for predicting injury risk related to repetitive muscle lengthening during learning and training of human complex motor skills. Springerplus.

